# A Set of Molecular Markers to Accelerate Breeding and Determine Seed Purity of CMS Three-Line Hybrids in *Brassica napus*

**DOI:** 10.3390/plants12071514

**Published:** 2023-03-30

**Authors:** Yanfeng Zhang, Ran An, Min Song, Changgen Xie, Shihao Wei, Daojie Wang, Yuhong Dong, Qingli Jia, Shuhua Huang, Jianxin Mu

**Affiliations:** 1 Hybrid Rapeseed Research Center of Shaanxi Province, Yangling 712100, China; zhangyfcl@126.com (Y.Z.); anrants@126.com (R.A.); songmin914@nwafu.edu.cn (M.S.); shihao100@126.com (S.W.); dyh9919@163.com (Y.D.); yljiaqingli@163.com (Q.J.); 2 State Key Laboratory of Crop Stress Biology in Arid Areas, Northwest A&F University, Yangling 712100, China; changen.xie@nwsuaf.edu.cn; 3 Key Laboratory of Plant Stress Biology, State Key Laboratory of Cotton Biology, School of Life Sciences, Henan University, Kaifeng 475004, China; wangdj@henu.edu.cn

**Keywords:** *Brassica napus*, cytoplasmic male sterile (CMS), molecular markers, seed purity identification

## Abstract

Cytoplasmic male sterility (CMS) is the main mechanism employed to utilize the heterosis of *Brassica napus*. CMS three-line rapeseed hybrids have dramatically enhanced yield and brought about the global revolution of hybrid varieties, replacing conventional crop varieties. Over the last half century, China has led the development of hybrid *Brassica napus* varieties. Two sterile lines, *polima* (*pol*) and *shaan* 2A, were of particular importance for the establishment of three-line hybrid systems in rapeseed, which has opened up a new era of heterosis utilization. However, in current breeding practices, it takes up to three years to identify the restorer or maintainer relationship and the cytoplasmic type of any inbred material. This greatly affects the breeding speed of new varieties and inhibits the rapid development of the rapeseed industry. To address this problem, we developed a set of molecular markers for the identification of fertile cytoplasmic gene N and sterile cytoplasmic gene S, as well as for the fertile nucleus gene R and sterile nucleus gene r, based on differences in the gene sequences between the CMS line, maintainer line and restorer line of *Brassica napus*. Combining these markers can accurately identify the CMS line, maintainer and restorer of both the *pol* and *shaan* systems, as well as their hybrids. These markers can not only be used to identify of the maintainer and restorer relationship of inbred materials; they can also be used as general molecular markers to identify the CMS-type hybrid purity of *pol* and *shaan* systems.

## 1. Introduction

The discovery of CMS was a milestone advancement in the development of global rapeseed cultivation, introducing a new era of heterosis utilization in *Brassica napus* (*B. napus*). More than 10 CMS types have been discovered in *B. napus*, among them *pol* [1], *shaan* [2,3], *ogu* [4,5], *nap* [6], *tour* [7], *nca* and *hau* [8]. The *pol* and *shaan* types belong to the same type of CMS system, sharing common fertility restorers [9]. In 1972, Fu et al. discovered the first male sterile line of rapeseed in the world, named *pol* CMS [10]. In 1985, Li et al. used the CMS line *shaan* 2A to breed the three-line hybrid variety Qinyou No. 2, which was the first widely promoted hybrid variety of rapeseed in the world [3,11]. Through over 40 years of continuous development, China has realized the hybridization of *B. napus* varieties, in which the *pol* and *shaan* CMS systems play a crucial role as the backbone of the sterile lines for the heterosis utilization of rapeseed.

CMS is a type of male sterility with a sterile phenotype controlled by the interaction between a cytoplasmic sterility gene S and a pair of recessive nuclear genes r; the genotype is named S (r r) [12]. Its maintainer with a fertile phenotype contains a cytoplasmic fertility gene N and a pair of recessive nuclear genes r, named N (r r) [12]. When the CMS plants are pollinated with pollen from the maintainer plants, the harvested seeds still show cytoplasmic male sterility. The restorer with a fertile phenotype contains a pair of dominant fertile nuclear genes R and has the two genotypes, S (R R) and N (R R). When the CMS plants are pollinated by the restorer, the harvested seeds are hybrids with an S (R r) genotype [12]. In breeding practice, the CMS line can be easily identified in the flowering phase by its extremely degenerate stamens. However, the identification of the maintainer and the restorer lines is difficult since both phenotypes generate normal stamens [12,13]. In order to identify the lines, they both need to pollinate the respective CMS line [13]. If all offspring are normal plants, the pollinated plant is a restorer, and if all offspring are sterile plants, the pollinated plant is a maintainer. If the pollinated plant produces both normal fertile plants and male sterile plants in equal amounts, it is identified as a heterozygous plant with an Rr genotype [13]. Plants with the Rr genotype cannot be used for breeding and generally account for about half of the total number, making this breeding system inefficient [12]. To identify the cytoplasm type of the restorer, a female restorer needs to be pollinated by the maintainer to produce F_2_-generation offspring [12]. If the F_2_-generation plants are all normal fertile plants, the restorer has N cytoplasm [12]. If the F_2_-generation results in about 1/4 sterile plants, the restorer has S cytoplasm. In general, it takes up to three years to establish the restorer and maintainer relationships of rapeseed plants [8]. This restricts the cultivation of new varieties. Therefore, developing a set of CMS specific molecular markers is of great importance to accelerate the breeding of new rapeseed varieties.

Through the application of molecular biology techniques, some AFLP (amplified fragment length polymorphism), RAPD (random amplified polymorphic DNA) and SSR (simple sequence repeat) molecular markers of *pol* CMS have been developed, and its restorer gene has been explored [14,15,16]. Furthermore, the molecular mechanism of *pol* CMS has been uncovered [1,17,18,19,20]. The mitochondrial genome of the *pol* CMS plant has an approximately 4.5 kb fragment more than that of its maintainer plant, which contains a specific open-reading frame, ORF224, upstream of the *ATP6* gene [18]. When co-transcribed with *ATP6*, it generates a new gene, *orf224-atp6*, which is largely responsible for the *pol* CMS formation [20]. Liu et al. located a PPR (pentatricopeptide repeat) gene in the restorer plant and proved that the PPR gene alone is the restorer gene of the *pol* system [1]. It is assumed that the post-transcriptional regulation of this gene cleaves the *ORF224*-*ATP6* gene, thereby degrading the ORF224 fragment and recovering male fertility [1,21,22]. Additionally, a functional molecular marker for the restorer of the *pol* system has been developed based on the restorer gene PPR. This marker achieves 100% reliability and is widely used for the identification of restorer plants and for the assessment of seed purity in *pol* CMS-type hybrids [23]. However, using only the restorer gene marker is not sufficient to accurately identify the restorer lines. The method is unable to distinguish between homozygous and heterozygous restorers, or between hybrids and their restorers. Therefore, it is necessary to develop a set of accurate and reliable molecular markers to identify the components of the *pol* and *shaan* CMS three-line hybrid systems.

In this study, we developed a set of molecular markers based on the gene sequence differences between the three lines of the *pol* CMS and *shaan* CMS *B. napus* systems. The markers are able to distinguish between sterile cytoplasm S and fertile cytoplasm N, and sterile nuclear gene r and fertile nuclear gene R. Using this set of molecular markers, the CMS lines of *pol* and *shaan* and their respective maintainers, restorers and hybrids can be identified accurately and quickly. The markers can be used not only to identify the restorers or maintainers of rapeseed materials, but also to assess the seed purity of *pol* and *shaan* CMS type hybrids.

## 2. Results

### 2.1. Developing Cytoplasmic Molecular Markers for N and S Cytoplasm Identification of the Pol and Shaan CMS Three-Line Hybrids

**Figure 1:**
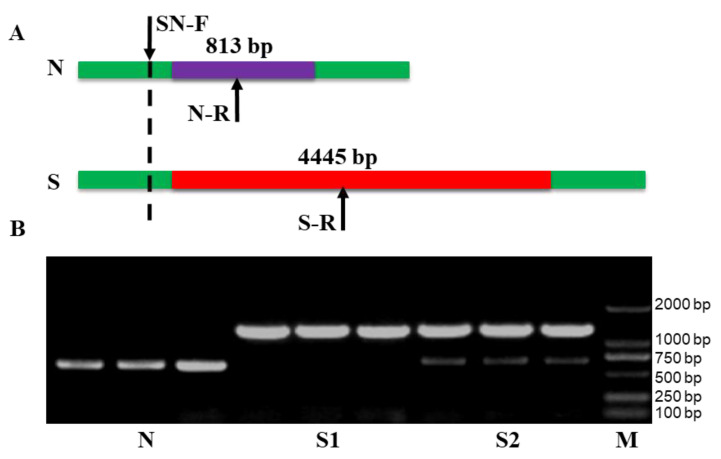
Identification of sterile cytoplasm S and fertile cytoplasm N of *pol-* and *shaan-*type CMS three-line hybrids by molecular markers. (**A**) The design schematic of S and N primers. (**B**) The identification results of S and N. The identical sequences of N and S genes are boxed in green. The inconsistent sequences of N and S genes are boxed in purple and red, respectively. N, fertile cytoplasm gene N; S, sterile cytoplasm gene S; M, DNA marker.

Mitochondrial resequencing of the CMS lines *pol* A and *shaan* 2A and their maintainers *pol* B and *shaan* 2B in *B. napus* revealed that the mitochondrial genomes of *pol* A and *shaan* 2A, unlike those of *pol* B and *shaan* 2B, both had the same exogenous fragment insertions, which were caused by a 4443 bp exogenous fragment replacing an 813 bp fragment on the mitochondrion of fertile cytoplasm N. Since there was no homology between the 4445 bp sequence and the 813 bp sequence, the specific reverse primers S-R and N-R were developed from the 4445 bp fragment and the 813 bp fragment, respectively. In addition, a common forward primer, SN-F, was designed for upstream of the fragment- replacement region (Figure 1A).

Using N- and S-type cytoplasm DNA as templates, PCR amplification showed that mixtures of the three primers, SN-F, S-R and N-R, could clearly distinguish between S cytoplasm and N cytoplasm; N-type cytoplasm amplified a clear 655 bp band, while S-type cytoplasm amplified a clear 1287 bp band (Figure 1B). In addition, S cytoplasm had two types of bands: the first one, which accounted for a very small proportion of the identified samples, had only one clear 1287 bp band, while the second one, which was the prominent type, had a clear 1287 bp band and a weak 655 bp band.

### 2.2. Developing Cytoplasmic Molecular Markers for N and S Cytoplasm Identification of the Pol and Shaan CMS Three-Line Hybrids

Based on specific single nucleotide polymorphisms (SNPs) of the sterile nuclear gene r and fertile nuclear gene R of CMS systems, the R-specific forward primer R-F and r-specific forward primer r-F were designed. At the same time, their common reverse primer Rr-R was designed. The R-F and Rr-R primers were used to identify the R gene, while r-F and Rr-R primers were used to identify the r gene. Because the amplification bands of R and r were the same size, PCR amplification and agarose electrophoresis for R and r identification were performed separately.

**Figure 2:**
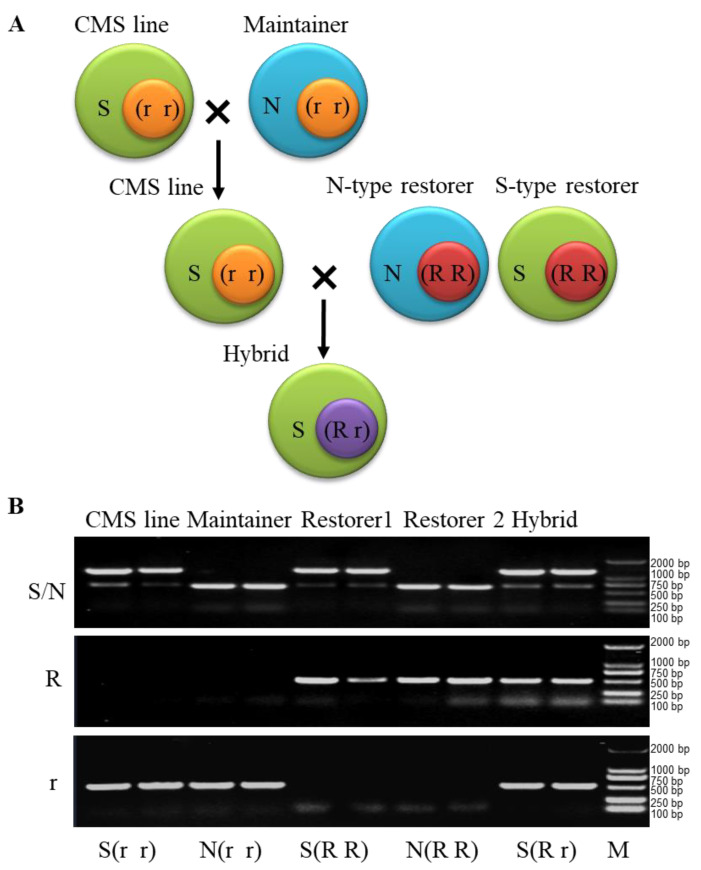
Identification of CMS three lines and its hybrids in *B. napus* by the molecular markers. (**A**) Schematic diagram of genetic pattern of CMS three lines and their hybrids in *B. napus*. (**B**) Identification of CMS three lines and their hybrids in *B. napus* by the molecular markers. N, fertile cytoplasm gene N; S, sterile cytoplasm gene S; R, fertility nuclear restorer gene; r, recessive nuclear sterile gene; M, DNA marker.

Figure 2 shows that all three lines (sterile line, maintainer line and restorer line) and their hybrids could be identified using PCR and a combination of the S/N, R and r molecular markers. The sterile line had S cytoplasm and only contained an r band; thus, it was identified as S (r r) genotype line. The maintainer exhibited N cytoplasm and also only contained an r band; thus, its genotype was identified as N (r r). Restorer 1 had S cytoplasm and only contained an R band and hence has an S (R R) genotype, while restorer 2 had N cytoplasm with only an R band and was identified as the N (R R) genotype. The hybrid, which had S cytoplasm and contained both an R band and an r band at same time, had an S (R r) genotype. These results are consistent with the genetic pattern of the three lines of the CMS system and reveal that the combination of S/N, R and r molecular markers can accurately identify the *B. napus* CMS sterile lines, maintainers and restorers, as well as their hybrids.

### 2.3. The Molecular Markers Are Suitable for the Identification of the Three Lines in the Pol- and Shaan-Type CMS Systems of B. Napus

The *pol* CMS and *shaan* CMS are two important systems for the heterosis utilization widely applied in rapeseed production [24]. In order to verify that our set of molecular markers can be applied in the identification of both CMS systems, we extracted the DNA of the pol (*pol* A and its maintainer *pol* B) and *shaan* (*shaan* 2A and its maintainer *shaan* 2B) systems as templates for PCR amplification.

**Figure 3:**
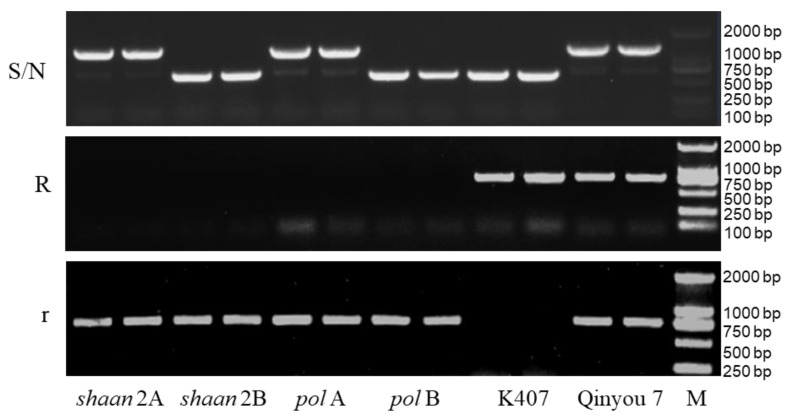
Identification of *pol* A and *shaan* 2A and their maintainers by the molecular markers. N, fertile cytoplasm gene N; S, sterile cytoplasm gene S; R, fertility nuclear restorer gene; r, recessive nuclear sterile gene; M, DNA marker.

The results show that the bands of *pol* A and *shaan* 2A both exhibited S (r r) genotypes. Similarly, the bands of *pol* B and *shaan* 2B both exhibited N (r r) genotypes. Thus, this set of molecular markers can be used as common markers to identify both the *pol* and *shaan* CMS systems simultaneously (Figure 3).

### 2.4. Identification of Rapeseed Germplasm with the CMS Three-Line Molecular Markers

**Figure 4:**
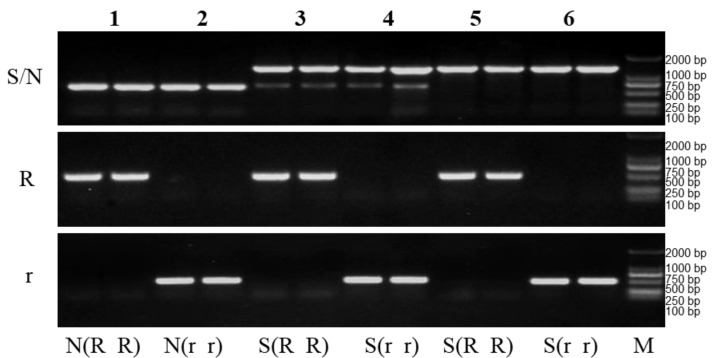
Identification of rapeseed germplasm with the CMS three-line molecular markers. N, fertile cytoplasm gene N; S, sterile cytoplasm gene S; R, fertility nuclear restorer gene; r, recessive nuclear sterile gene; M, DNA marker. The Arabic numbers (1 to 6) indicates different types of the three CMS lines: CMS sterile lines (samples 4 and 6), maintainers (sample 2) and restorers (samples 1, 3 and 5).

In order to verify the accuracy of this set of molecular markers, the CMS sterile lines (samples 4 and 6), maintainers (sample 2) and restorers (samples 1, 3 and 5) were taken and examined in the flowering stage. PCR amplification confirmed that samples 4 and 6 were of the CMS genotype S (r r), sample 2 was of the maintainer genotype N (r r), sample 1 was of the restorer genotype N (R R) and samples 3 and 5 were of genotype the S (R R) (Figure 4). These results are consistent with the known phenotypes of the test samples. They also clarified the S or N cytoplasmic type of the restorers. Thus, these markers can be used for the fast and accurate identification of rapeseed germplasms.

### 2.5. Identification of Rapeseed Germplasm with the CMS Three-Line Molecular Markers

The rapid and accurate identification of seed purity is of great importance to reduce the risk of low-quality seeds, to ensure seed safety and to safeguard farmers’ rights and interests. In the past, it usually took one year to identify seed purity through field planting. In order to shorten the identification time, molecular markers such as RAPD and SSR have been applied to the identification of hybrid purity [25,26]. However, these markers usually only correspond to a specific variety, and the change in variety requires the re-development of molecular markers. At present, the *pol* and *shaan* CMS-type hybrids still represent the majority of rapeseed in China [12]. This set of CMS molecular markers can be used as universal markers to identify hybrids purity.

**Figure 5:**
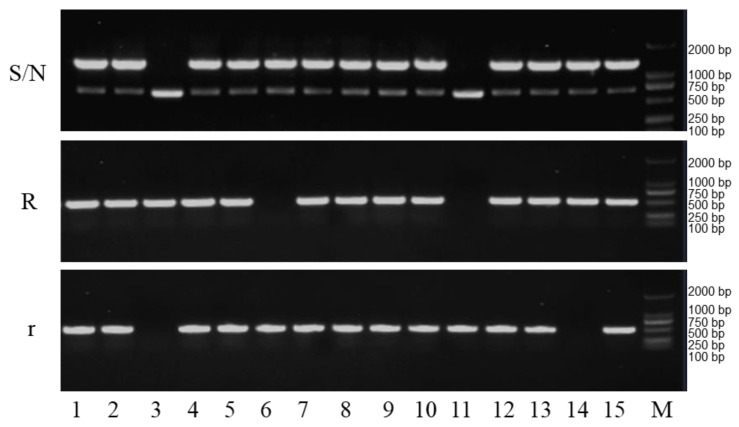
Identification of seed purity for CMS three-line hybrids with the CMS three-line molecular markers. N, fertile cytoplasm gene N; S, sterile cytoplasm gene S; R, fertility nuclear restorer gene; r, recessive nuclear sterile gene; M, DNA marker. The Arabic numbers (1 to 15) indicates different hybrid seedlings.

DNA samples extracted from hybrid seedlings that were germinated for at least four days were used as templates for PCR amplification. The analysis of the results shows that two samples (3 and 14) were restorer seeds, sample 6 was a sterile seed, sample 11 was a maintainer seed and the remaining samples were hybrid seeds (Figure 5). The proportion of hybrids, sterile lines, maintainers and restorers in the test samples was 73.3%, 6.6%, 6.6% and 13.3%, respectively.

In conclusion, these molecular markers can be used to identify the germplasms and the seed purity of *pol* or *shaan* CMS-type hybrids. For seed purity, the entire identification can be performed in less than one week, which greatly shortens the process time and ensures the quality and safety of rapeseed hybrids.

## 3. Discussion

The identification of the relationship between the maintainer and restorer in rapeseed is crucial for cultivating new varieties, which is directly related to transferring a sterile line or breeding a restorer [1,27,28,29,30]. In this study, the S and N molecular markers developed are derived from the indel differences in mitochondrial DNA between fertile cytoplasmic N and sterile cytoplasmic S, while the R and r molecular markers developed are derived from core SNP differences between the restorer gene R and the sterility gene r in the nucleus [12,31]. The combination of these molecular markers can rapidly and accurately identify the CMS lines, maintainer lines, restorer lines and hybrids of the *pol* and *shaan* CMS systems [2,31]. This can be applied to the identification of both the maintainer or restorer relationship in inbred lines, and the rapeseed purity of the cytoplasmic three-line rapeseed hybrids. This is of great significance for accelerating the cultivation of rapeseed varieties and ensuring the seed quality and safety. For seed purity, the entire identification can be performed in less than one week.

Although this set of CMS three-line molecular markers can accurately identify the purity of rapeseed inbred lines and its hybrids, attention should be paid to obtaining accurate and reliable identification results by strictly following the described experimental protocol. Inconsistent DNA concentrations will lead to unstable PCR bands, resulting in inaccurate identification results. The Taq enzyme is also an important factor affecting the identification results. There is only a slight difference in the three bases between the specific molecular markers of the nuclear sterile gene r and the nuclear restorer gene R. When amplified by the common Taq enzyme, some restorer samples (which should not have any bands) will show a light band, which is significantly different from the clearly visible R band. From experience, we know that this light band is an artefact. If amplified by some high-fidelity Taq enzymes, the brightness of this non-specific band will increase, which can cause misinterpretation of the result. Therefore, we recommend that the MIX products with common Taq enzymes be used as much as possible during the identification process.

CMS-type hybrid systems usually consist of three types of seeds: the hybrid seed; the sterile seed, which is a serious negative factor affecting the purity of hybrids and must be removed [12,27]; and the restorer seed. The maintainer seed is rarely mixed into hybrids and is generally ignored. Hence, in order to improve identification speed and save costs, the identification of S- and N-type markers can be omitted, and the R and r molecular markers are sufficient to determine seed purity. However, if seed purity is too low and needs to be re-established, we suggest using S/N molecular markers to determine whether the reason for the impurity is present because the sterile line is mixed with maintainer seed or the restorer line is mixed with (R r) heterozygous plants. If the low purity is caused by the impurity of the parents, the parents can be purified according to the identification results to improve the quality of subsequent seed production. Overall, this set of markers has strong applicability and important application value and can greatly shorten the time required to identify the restorer and maintainer relationships of rapeseed plants. Furthermore, it can also be used as a universal marker to identify the purity of *pol* and *shaan* CMS three-line hybrid, which is important in ensuring the seed quality and safety of hybrids.

## 4. Materials and Methods

### 4.1. Materials

The rapeseed CMS lines *shaan* 2A [2,3] and *pol* A [1] of rapeseed and their maintainers *shaan* 2B and *pol* B [3] were sourced from Dianrong Li. The rapeseed CMS hybrid Qinyou 7 of rapeseed and its sterile line *shaan* 3A, its maintainer *shaan* 3B and its restorer K407 were sourced from Dianrong Li. Some other rapeseed inbred lines and three-line hybrids, also sourced from Dianrong Li, were used for identification.

### 4.2. Protocol for High Throughput Screening and Rapid DNA Extraction for Rapeseed

We added one stainless steel ball (5.0 mm diameter) into each hole of a 96-well medium-hole plate (30 mm height). For each plant sample, we took one piece of round leaf using a hole puncher with a 3 mm aperture. We loaded each sample into a matching hole using tweezers and filled in the sample register (Figure S1). We covered the plate with a silicone cover and transferred it into an ice box with a temperature of 4 °C. Samples were either directly taken to the lab for DNA extraction or stored at −20 °C. The seed DNA was extracted after germination for at least 4 days. For each sample, 200 µL 1 × DNA extraction solution (10 mmol·L^−1^ Tris (pH 9.5), 0.5 mmol·L^−1^ EDTA and 0.1 mol·L^−1^ KCl) was added into the well, and we covered the plate with a silicone cover. The plate was then put into a grinder (Tissuelyser-192, Shanghai Jingxin), where the samples were ground for 2 min at 30 Hz, repeated once. The plate was then placed into a centrifuge (Eppendorf 5810R with four plate buckets) and the samples were centrifuged for 5 min at 3000 rpm. The plate was stored at 25 °C for 1 h or overnight at 4 °C. The supernatant was then used as a template for PCR amplification.

### 4.3. Primer Design

Sequence analysis of cytoplasmic genes of the CMS lines and their maintainers was performed using the Vector NTI software. According to indel (insertion-deletion) differences in mitochondrial DNA between S cytoplasm and N cytoplasm, three primers, SN-F, N-R and S-R, were designed using the Primer Premier 5 software (Table 1**,** Figure S2). The cytoplasmic fertile gene N was identified using the SN-F and N-R primer pair with a 655 bp amplification band, while the cytoplasmic sterile gene S was identified using the SN-F and S-R primer pair with a 1287 bp amplification band. Due to the sharp difference in size between the amplified N and S fragments, S and N genotypes can be clearly separated when mixing all three primers into the same reaction system for PCR amplification.

To identify the sterile nuclear gene r and fertile nuclear gene R among the CMS three-line hybrids, we designed the R-specific primer R-F, the r-specific primer r-F and a common reverse primer Rr-R based on specific single-nucleotide polymorphisms (SNPs) (Table 1, Figure S3).

### 4.4. PCR Reaction System and Procedure

PCR reaction system (16 µL): 1 µL DNA template, 8 µL 2 × Es Taq Mix (CW0690, CW Bio), 6.0 µL ddH2O, forward and reverse primers, each 0.5 µL. For S/N identification, add 5.5 µL ddH2O, SN-F, N-R and S-R 0.5 µL, respectively.

PCR procedure: Pre-denaturation at 95 °C for 5 min, denaturation at 94 °C for 40 s, annealing at 60 °C for 40 s, extension at 72 °C for 60 s, cycle 30 times, final extension at 72 °C for 10 min and preservation at 16 °C. In the present study, the Tm value of N/S and R was at 60 °C, while that of r was at 61 °C.

Electrophoresis: PCR products were detected using 1% agarose gel electrophoresis, 140 V, 15 min. DM2000 DNA ladder (CW0632, CW Bio) was used in the present study to determine the size of the DNA fragment. The gel was stained using ethidium bromide solution (1.0 mg/100 mL). Photographs were taken with the gel imaging system (Bio-Rad).

## 5. Conclusions

In this study, a set of molecular markers for both *pol* and *shaan* CMS three lines of *B. napus* were developed. Combining these markers can accurately and rapidly identify the CMS line, maintainer line and restorer line of both the *pol* and *shaan* CMS systems, as well as their hybrids. These markers can not only be used to identify the maintainer and restorer relationship of inbred materials; they can also be used as general molecular markers to identify the purity of *pol* and *shaan* CMS-type hybrid systems, which is important in ensuring the seed quality and safety of hybrids.

## Figures and Tables

**Table 1:** Primers of molecular markers for identification of *pol* and *shaan* CMS three-lines as well as their hybrids in *B. napus*.

Primer Name	Sequence (5′→3′)	Base Number
SN-F	TTCATACGGCGAGAGTCATTG	22 bp
N-R	CAAGACCATAGAATAGGAGAACCAC	22 bp
S-R	GCTCGTTCGGCTACTTATCTTG	22 bp
R-F	GGGATGCGATCCTGATATTTG	21 bp
r-F	GGGATGCGATCCTGATATCG	20 bp
Rr-R	CTCCAAAAGGACCAGAAAGCA	21 bp

## Data Availability

Not applicable.

## Supplementary Materials

The following supporting information can be downloaded at: https://www.mdpi.com/article/10.3390/plants12071514/s1, Figure S1: Schematic diagram of the sample loading; Figure S2: The nucleotide acid sequence of N and S regions; Figure S3: The alignment of R and r nucleotide acid sequences.
