# Supplementary material for: A Set of Molecular Markers to Accelerate Breeding and Determine Seed Purity of CMS Three-Line Hybrids in Brassica napus
Source: Plants (Basel). 2023 Mar 30;12(7):1514. doi: 10.3390/plants12071514 (PMC10096742; doi:10.3390/plants12071514)
Supplement: Supplementary file 1 [file plants-12-01514-s001.zip › plants-2295240-supplementary.pdf]

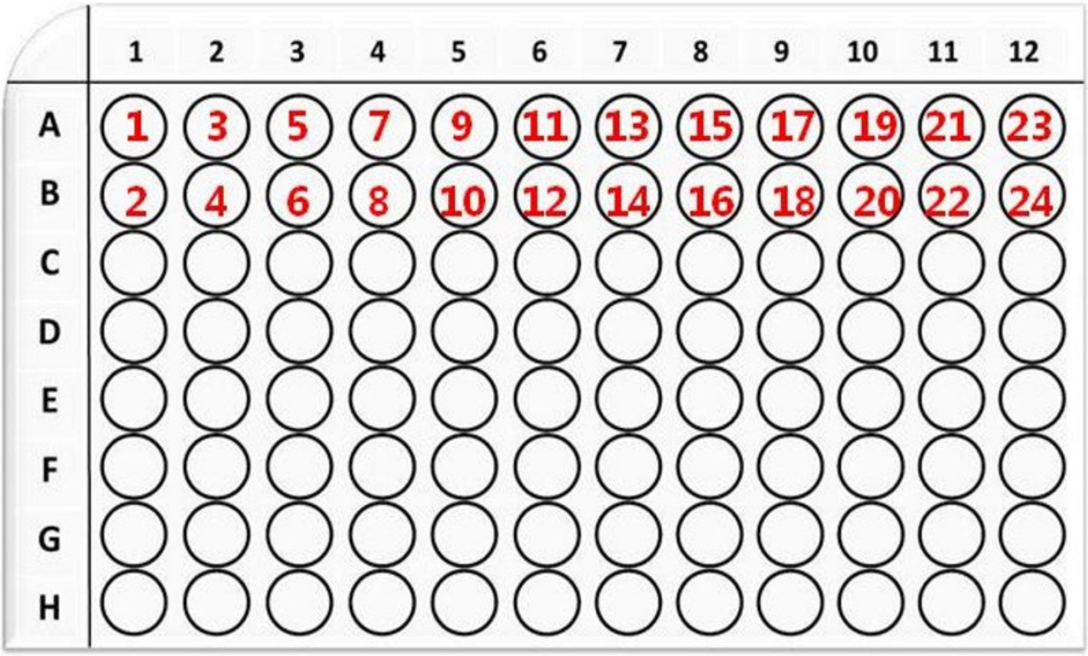

Figure S1. Schematic diagram of the sample loading.

N (KP161618.1):

GGCGCTCTACCTCTGGCTATGCTATTTTTCTTGGTCCCAATCTTATTTCATGGCGCGCC
AAGAAAGAACCTACAGTCTCTCGTTCTAGTGCTGAGTCTGAATACCGCTCGCTTGCCT
TTGCTGTTGCTGAGTCTCACTGGATTACTCAGCTACTTCGCGAGCTTTGTCTCTTTCTTC
CTCACCCTGTTCAGATCTATTGTGATAACATCAGAAAACTTATATGACTGCCAATCCT
GTTCATCATGCTCGCTCGAAACATATTGAAATTGACTACCATTTTGTACGGGAAAAGG
TTGTCAAAGGTGACATTATTGTGCAATATATTCCCACATCCGAGCAACTTGCTGATAT
CTTCACGAAGGGCCTCCCCTCTGCCCAATTTCACTACCTTCGCTCCAATCTTCGCATCC
TTCCACCTTGCTCCGATTGAGGGGGAGTCTTAGTGTATCTAGGTTATACTGTTAATTGT
GTCGTATGTATTAGCCCAAGGGCTTAATTGTAATTTGACTTGAAGAGATCATCTATAT
AAATAGACATCTGATGCCCTAATTAGGGCTAAGCACTGAAATTTGTCCTAAACCAATT
TCAGAAACATTTTGTTATGGTGGGGAGTGAAAACACCAATGCAGCAGAGAACGACC
GTATTAATCGGAATCTACTTATATTAAGACAGACGACCGAGAAAGAAAAGAAAGGC
TCCACGTTCTCCAAGTGCTTAATCTTAGCGTGCTAGCCGCTGCTTCATTTCGTTTTCGT
ATAGTGAGAACGCTTTCTCTTTCTTAGCGCTCCGCACCCTTGTATAGTAAGGGGAACT
CTGGTTGCGCGGCTTTCTCTTATAGGGGTCTATTTTCTCTGACTTGACTCAGCTTGACCT
ACTTGACTCAGCGGTTAGAGTATCGCTTTCATACGGCGAGAGTCATTGGTTCAAATCC
AATAGTAGGTAAAACCGGCCGAAACCCCTGCTTTTTCCAGCATGACAGCAAGCACGG
ACTGGCAGCAAGGATGAAACTGAATGACCAAAGCATCGGTTGCTTCCACTTGTGGCC
TTCTTCGACCGCCTTGACACCTTACTTAATATCAAGGCAGCCCACATACATGAAGAGA
AGAATTGGGTTTAGCGGTAATGGCATAAGCCAAAGCTAGATCACCGGAGTTTAGGCA
AATGGGTACGAGAGAAAAAGGTTTTGAAATGCATCTTCCGAAACCAGATAATCAACC
GAAGGAGATTCCCCTGCAAAAAGGAGATGGCTCTAGTTTCATACTAATCTTCGATCTT
CGAGATTCCCCCACGGAGCGGTAAGGCATCCCAAGGAGCGAAGCGGCTCAAAGGAT
TGGCAATGAAGCTCAGTCTGGTCTGAGGGGATGGGCTAGGCTACTCTCCCCATACAT
AGGAAGAGGGGGAAGTCAAGTACCTAGATCCAAGATCACGATATCCGCTTTGGTGGT
AATATCCGGCATAAAAGAAAGCACCAAAGCAAACAAAGTCGATTCTTTGTTGAGTCA
ACTGGCAACTGGGATTGCAGCGGATCTAGTGGTTCTCCTATTCTATGGTCTTGCTGCTG
AGAGAGTAAGAATAGAAAGCAGAAGCTCGCCCGCGGGCGAAAGACAAGGGATGAT
TCTTCTAAGATACGAACTTGCGGAAGGAATGAAAAGGTATTCGACTAAGACGGGGAC
GGGTATTCCCGAAGATGCTTGACCTAAGAGATAGAGACGAGAACGAGACTAAGAAA

ATCAGATATTGGAGAGGAAAGAAGCAGTGCGAGTAGAGTAGAGCAGCTTGGTAGCT
CGCAAGGAGCGAAGCCTGCTTGACCAACGGGAGAAGCCAACTCCGTTCCTCCAGCTT
CGCTGAAGAAGCTAGGTTCCTCCTTGATGTCGTAGGTTCAAATCCTATCTCCGCACTA
AGTAAGGGTTTCATTCTGCATCACTCTCCCCGTCGTTCTCGACCTCGCAAGGTTTTTGA
AGCGGCCGAAGCGGGAAGTGACAATACCGCTTTTCTTCAGCACATTTTGGATGATTTG
AGCGAAAACGGAGTACAAAGTTCAGCCTTTAAGGAGGCTATGAATCAAATAGGGCT
GGTGGCGCAGTCCCCACTTGACCAATTTGAGATTGTCCCATTGATTCCTATGAATATC
GGAAACTTCTATTTCTCATTCACAAATCCATCTTTGTTCATGCTGCTAACTCTGAGTTTT
TTCCTACTTCTGATTCATTTTATTACTAAAAAGGGAGGAGGAAACTTAGTCCCAAATG
CTTGGCAATCCTTGGTAGAGCTTCTTTATGATTTCGTGCTGAACCTGGTAAAGGAACA
AATAGGTGGTCTTTCCGGAAATGTGAAACAAATGTTTTTCCCTTGCATCTTGGTCACTT
TTCTTTTTTTGTTATTTTGTAATCTTCAGGGTATGATACCTTATAGCTTCACAGTGACAA
GTCATTTTCTCATTACTTTGGCTCTCTCATTTTCTATTTTTATTGGCATTACTATAGTGGG
ATTTCAAAGACATGGGCTTCATTTTTTCAGCTTTTTATTACCCGCAGGAGTCCCACTGC
CGTTAGCACCTTTTTTAGTACTCCTTGAGCTAATTTCTTATTGTTTTCGCGCATTAAGCT
TAGGAATACGTTTATTTGCTAATATGATGGCCGGTCATAGTTTAGTAAAGATTTTAAG
TGGGTTCGCTTGGACTATGCTATGTATGAATGAGATTTTCTATTTTATAGGGGCTCTTG
GTCCTTTATTTATAGTTCTTGCATTAACCGGTCTGGAATTAGGTGTAGCTATATTACAA
GCTTATGTTTTTACGATCTTAATCTGTATTTACTTGAATGATGCTATAAATCTCCATTAA
AGTTCTTCTTTCTTTTATTTAGATTT

S(FR715249.1):

GGCGCTCTACCTCTGGCTATGCTATTTTTCTTGGTCCCAATCTTATTTCATGGCGCGCC
AAGAAAGAACCTACAGTCTCTCGTTCTAGTGCTGAGTCTGAATACCGCTCGCTTGCCT
TTGCTGTTGCTGAGTCTCACTGGATTACTCAGCTACTTCGCGAGCTTTGTCTCTTTCTTC
CTCACCCTGTTCAGATCTATTGTGATAACATCAGAAAACTTATATGACTGCCAATCCT
GTTCATCATGCTCGCTCGAAACATATTGAAATTGACTACCATTTTGTACGGGAAAAGG
TTGTCAAAGGTGACATTATTGTGCAATATATTCCCACATCCGAGCAACTTGCTGATAT
CTTCACGAAGGGCCTCCCCTCTGCCCAATTTCACTACCTTCGCTCCAATCTTCGCATCC
TTCCACCTTGCTCCGATTGAGGGGGAGTCTTAGTGTATCTAGGTTATACTGTTAATTGT
GTCGTATGTATTAGCCCAAGGGCTTAATTGTAATTTGACTTGAAGAGATCATCTATAT
AAATAGACATCTGATGCCCTAATTAGGGCTAAGCACTGAAATTTGTCCTAAACCAATT
TCAGAAACATTTTGTTATGGTGGGGAGTGAAAACACCAATGCAGCAGAGAACGACC
GTATTAATCGGAATCTACTTATATTAAGACAGACGACCGAGAAAGAAAAGAAAGGC
TCCACGTTCTCCAAGTGCTTAATCTTAGCGTGCTAGCCGCTGCTTCATTTCGTTTTCGT
ATAGTGAGAACGCTTTCTCTTTCTTAGCGCTCCGCACCCTTGTATAGTAAGGGGAACT
CTGGTTGCGCGGCTTTCTCTTATAGGGGTCTATTTTCTCTGACTTGACTCAGCTTGACCT
ACTTGACTCAGCGGTTAGAGTATCGCTTTCATACGGCGAGAGTCATTGGTTCAAATCC
AATAGTAGGTAAAACCGGCCGAAACCCCTGCTTTTTCCAGCATGACAGCAAGCACGG
ACTGGCAGCAAGGATGAAACTGAATGACCAAAGCATCGGAAAAGCAGAGGAGTGTG
CTGACTTCAGTAGCTAGTCAACCAACTTCTGCTCAACCCTAGGAACAAGGAAAGTAT
CAGCCGATAACTCTGTTCACTTGGTGTGGGATTCCTAAAAGTTTACTATTATCGCACG
CCAAAGAGTGTGCTGAAAAGGGAATAGTTGGTTAACTACCGAAGCTAGAAACAAAT
ACCTGCTAAAACCAAGGGAAGCAAGAGTAGGAACTACCCACCCAGGAATCAGCATT
CTAAACTGCAGCACAAGCACAACCCTGCTTTTCCATTCCTTCATAGCCCACCTCTTCA
TTGGCCATAAAGCCTTGATTTCCTTGGACCACGTACACTGATGATTGGTCGGGCTGGG
CTACTCTATGGGCGTATGCGCCTAAACCAGGCTCAAAGCGATGAGACTTCTCGGTTA
GACTCAAACAATAGATCAGGTCAGTTTTGAGGAGCTTTATTTGACTTAAGAAGAAAG
GGCAGCCCTCTGATATATCTTTCGACTTCACCCTCGGCCAAAGCACAGTAAGTTAGAG
ACTCCTAGTCTCTACTAAATCAATCTATAGCTTCAACACTCTCCTAGCGCAGCAACCT
TCTCTCTTTCTCCCTTACTTGCCTCCCAGTCTTGAGCAGCAATCATCTTTGCTTATGCGG
ACGAAAGTCTTTCCACAGCACTCCGACAGAAGTCTCATATGATGACTCTGTTTACTCA
AGAGCCTTCTTTCTCGATTAAATGCCTGCCCACTACCCCAAGCAAGCCGCATAGGTCT
TTGTTAGCAGCTAGGCTTCTGTTTCTTTCCATAAGCGACTTCTCCTGTAGATGCCCAAA

ATAAAGCTTTCGCTCAAGGGATATCGCTTCATTTACTGCGTTAGGGTGGTGCCAAGCC
CTTCATCTCTGGCTAAGGCTCAGTCAGATAGGCTCAATATCGGGCTTATCCGGCGAAG
TCTCAAACTCTGTTGTGCCAGGGATATCAGGCTTGAGTTGGGACAGGTGCTTATTTCG
ATACCGCTTCTGTTTGTTGTTTTTGTGATGCTAGTGATCACATTTACAGTTAGAAGTAG
GCCATGATTGATTAGCTAGAGTAGAGCTAGGTTGGTGCTGCTTTGCTTGGCAATCCTT
GATCTAATTGAATCAGAATCCGCAAGATAAGTAGCCGAACGAGCAGAGCAGTAGAA
GGACAGGTCATCTAGAATCCTATTCTTCCGATGCTTTGATCGGTTCCTTCTGATATGGA
AGAGTTGACTACCCAGCACATCTTTGTTTGCTCGACTCGGTAAGTACAGCCGCTTCCT
TTATTCTTTTCTTTGAACCATTACTCTCAAGGGACTACTCCGTTGGCCCAGTGAACCTT
GGTCTTTTGTTGCTGTTCCCCGTTCCCTTTATCAGCTCTTTACCAGCTATCCGATTACCA
ATGAACAATAGCTATGACCGATAACCAAGGTTCGTAGAAACTCTATAAGGAGAGGC
ATAGACCGGTGGTCCCTTCCAAGTGCCTCTTTGTTTTGTACGAGCTGTTTCCAAGTGTT
TTGATTAGTCGAGTCTTCTTCTGAGTCCAGCACTTTTCCTTTGTCCGATCGTCCGTCTCT
TCCTCTCCTCTTGGTCGAGTTGCTAAAGCACCTATCTTTCTGATCCGAAGCCAAGGGA
TGTGCGATGTTTCTTTCTTTTGTTATTGTGCTAATTGCACTTTAGATTGAGAAGATAGG
ACTGATCGGTATCCATTGAAGATTCCCTTGCTTATCGGCATTCCTGCCCTGTGCACTGC
TTCCTCAGCTTCGAAGCTTGGTTTGTTTGTCTCGAAGATTGACGACTTATCGGCTGTTG
ATTGACCGGTATCTGGTATGCTTCTTCTTCCTTACAGTCGACTGTTGCTACTTCGGACT
ACTACCATTCACTGCTGAGAAGAAGCTGTTGTTTAAGGAGCGGCACCGATCAGTGCC
TTGTTCCTGTTACCAGTGTCTTGTCTTGGTTCCTTGTGCCCATTGTCTCCTAGTGTTTTGT
TGAGTTTACTGTGTCGATAAAGTCCTTTTTATTCGAGGAAGTGTAGCCTTTTCAATTAC
AATTCTTTTATTCGTCCGAGTTTACTGTGCCATTTCGAGACTGTCCTTCATCCGAGGTA
TCGTGTTTATTGTGCCATTTATAGACATTGAGGACATTACTGTGTTGTACCAAGCACCA
ATAGTGTGAGGATTAAGCCGTTATTCTATCTTCCTTCCTTCGCTCTAACGGGTGGATTC
GCTCTAACAGGAGGATTCCGCTCACTTGGGGTGGAGTAGCGTATGCACCTCTCTTTCT
TTGTTGCTTCCGGTGTGCTACCGAGCCTTTCCCAGGTATTTACTTACTGCCGGTTTACTC
CACAGCATTGCCATCTTCTTTGTTTTGTTGGTATGAGCTGATCGGCCCTCTCAAGCGTA
AAGTAGTCCTCTGATTGTGTTGTATTGGCTCCTTAAGCAACATTTGTTTACTTATGACC
GGGTGCTGCTTATGCCTAGACATCTTCCCCAGGACTTCTTCTCGAAAGACGTTGACCT
ATGGTAGGTGCTGAGTCCAGCACAGTTGTTCCTTTGTTACTGTGCCATTTTGAGACCTT
CGTCCGATCATTGTTCCTTTGAAGACCTGTCTTTTATTCCTTCTCTTTCCTTGCAGTAAT
TAACTCACATTTATGGTAATGCTGATGCTTGGCAGCTTCACTGTTCCTTCTTGTTGTGC
GTAGGCTTGGCGTTGTTTACTCCACCGTGCTATCTTTGTGCTGCTAATCAGTATAGTAT
ATTCTTTGCATACTGCTATCTTTGTGCTAATCCATTTCAGTAAACGGGAAAGTGGAGG
AGTAATGCTTGGTCAAAGTGCTGTAGATTTGTTTCGGTACGAGCACTGCCTTTGTGCTA
ATCATCATTTCAGTATATTCTTTCTTTCGTGTTCCATCCTGATTAATCTGTAATCTATGT
TTTGTACAGCTGGCTTGGGAATCATCAGTAGAGTCTTTCTGCTCCTGATCTGTCAACTT
TCTTTCGTACGGCTGCTTGGCGTGCTGTCATCTTTGCTACTCCGCTTCGTACGGTTTTGG
GTGAAGGTTCAATCTTTTGTTTCGTACGAGTCGGATATGTTTGTGCGAGAAGAAGGAC
AGCTTTCGTAGAGAGTCCGATCAATCTTCCACTCTTGCACTTGCATTCATTCGGTTCTC
CTCTCAGTTCCGTCTAAATCCCCTCGTCCTGACTAAAGCACCTCTCCGTGTTCCGTCGA
TCACCTAGATAAATTAAGTATAGTAATAAGAATGAAAAGTATGGCTTAGCGTGTTGG
ATCAATCAGTCATTGCTTTCATCTTGAATTAAGTATATAGAAAAGAAGTGTTGTCTGC
GCCAAGTCTAGAAGTGGTAGTATTAGCGATCCTATTGAGGTGTTTATCGGGTTCGCTG
CTCGAATTTCAATAAAGTCAAGTTTCCTCAATCCCGTTTCTGTTGAGTTGCCAAGATTT
CTCAATCCTTGTATTAGTTGATCCTTTTGTTTATTCGAAAGGTCGGCGGGATGCTACTT
CAGGTAGGGTACGGGCGCTCTATCATTGTCTGATTTTAGGTTTCTGATCGCTAGCCTGC
CGGGCTGCCCCCGCGATCAAACTATCAATCTCATAAGAGAAGAAATCTCTATGCCCC
CTGTTCTTGGTTTTCTCCCATGCTTTTGTTGGTCAACAACCAACCACAACTTTCTATAGT
TCTTCACTACTCCTAGAGGCTTGACGGAGTGAAGCTGTCTGGAGGGAATCATTTTGTT
GAAATCAATTAATCTAATCATGCCTCAACTGGATAAATTCACTTATTTTTCACAATTCT
TCTGGTTATGCCTTTTCTTCTTTACTTTCTATATTTTCATATGCAATGATGGAGATGGAG
TACTTGGGATCAGCAGAATTCTAAAACTATGGAACCAACTGCTTTCACACCGGGGGA
AGACCCTCCTGAGCAAGGGAAGGCTTGGAAAAAATCGTAGTTCAGATTCAAGTCGGT

TCGAGGTATCAGCGTTGGCCGCCCATTATTTTATCATTTTCGTGGTCCCAAAATTGGGA
CCAGTTTTCTACATTATATATAATTTTTTTTGTTTGTTGGGGTTGAAATGGGGGGTATTA
GGAAATGAAATTTGTCATTTCGGCGTCGGACCAGATGGCGTCGCGCCCCCAGCGCTG
GATCTCAACGAGCGCCCGCCTCTGCATCTTTTGTACGCGGATGTTGAGAGTTCCGACT
CTCAACAAGCGCGAAATAATGACATGTACGCGCATCTTAGGCGCGTACAGGAGATCA
CCCAAAAACTAGAGGGTGAGCGCGATATCGTGCGGCGTCAAGCCCTCCTGGATATAA
TGAAATGGGAGGTCAGAAGCCTTCAGGAGCACTTTCGGATCTTTCGGCACCTTGATCG
TCTGCGAGATTCGCAGAGAGCCAAGGTGAACGAGATCCTTGATCTCTTTCGCTGAAG
AAGCTAGGTTCCTCCTTGATGTCGTAGGTTCAAATCCTATCTCCGCACTAAGTAAGGG
TTTCATTCTGCATCACTCTCCCCGTCGTTCTCGACCTCGCAAGGTTTTTGAAGCGGCCG
AAGCGGGAAGTGACAATACCGCTTTTCTTCAGCACATTTTGGATGATTTGAGCGAAA
ACGGAGTACAAAGTTCAGCCTTTAAGGAGGCTATGAATCAAATAGGGCTGGTGGCGC
AGTCCCCACTTGACCAATTTGAGATTGTCCCATTGATTCCTATGAATATCGGAAACTT
CTATTTCTCATTCACAAATCCATCTTTGTTCATGCTGCTAACTCTGAGTTTTTTCCTACT
TCTGATTCATTTTATTACTAAAAAGGGAGGAGGAAACTTAGTCCCAAATGCTTGGCAA
TCCTTGGTAGAGCTTCTTTATGATTTCGTGCTGAACCTGGTAAAGGAACAAATAGGTG
GTCTTTCCGGAAATGTGAAACAAATGTTTTTCCCTTGCATCTTGGTCACTTTTCTTTTTT
TGTTATTTTGTAATCTTCAGGGTATGATACCTTATAGCTTCACAGTGACAAGTCATTTT
CTCATTACTTTGGCTCTCTCATTTTCTATTTTTATTGGCATTACTATAGTGGGATTTCAA
AGACATGGGCTTCATTTTTTCAGCTTTTTATTACCCGCAGGAGTCCCACTGCCGTTAGC
ACCTTTTTTAGTACTCCTTGAGCTAATTTCTTATTGTTTTCGCGCATTAAGCTTAGGAAT
ACGTTTATTTGCTAATATGATGGCCGGTCATAGTTTAGTAAAGATTTTAAGTGGGTTCG
CTTGGACTATGCTATGTATGAATGAGATTTTCTATTTTATAGGGGCTCTTGGTCCTTTAT
TTATAGTTCTTGCATTAACCGGTCTGGAATTAGGTGTAGCTATATTACAAGCTTATGTT
TTTACGATCTTAATCTGTATTTACTTGAATGATGCTATAAATCTCCATTAAAGTTCTTCT
TTCTTTTATTTAGATTT

Figure S2. The nucleotide acid sequences of N and S regions.

```
                                1                                                                              80
KX671969  rapa Rf     (1)   ATGGTGTTGAGGACACAGAGATGGAATCGTCTTACTACTTTGAGATTGGTTCATCTCCGTTCAACTGAGACAGGTACTCT
KX671968  rapa Rf     (1)   ATGGTGTTGAGGACACAGAGATGGAATCGTCTTACTACTTTGAGATTGGTTCATCTCCGTTCAACTGAGACAGGTACTCT
KX671967 rapa Rf      (1)   ATGGTGTTGAGGACACAGAGATGGAATCGTCTTACTACTTTGAGATTGGTTCATCTCCGTTCAACTGAGACAGGTACTCT
EF584011 Rf           (1)   ATGGTGTTGAGGACACAGAGATGGAATCGTCTTACTACTTTGAGATTGGTTCATCTCCGTTCAACTGAGACAGGTACTCT
KX671970 rapa rf      (1)   ATGGTGTTGAGGACACAGAGATGGAATCGTCTTACTACTTTGAGATTGGTTCATCTCCGTTCAACTGAGACAGGTACTCT
KX671971  rapa rf     (1)   ATGGTGTTGAGGACACAGAGATGGAATCGTCTTACTACTTTGAGATTGGTTCATCTCCGTTCAACTGAGACAGGTACTCT
KX671972 rapa rf      (1)   ATGGTGTTGAGGACACAGAGATGGAATCGTCTTACTACTTTGAGATTGGTTCATCTCCGTTCAACTGAGACAGGTACTCT
KX671973  rapa rf     (1)   ATGGTGTTGAGGACACAGAGATGGAATCGTCTTACTACTTTGAGATTGGTTCATCTCCGTTCAACTGAGACAGGTACTCT
KX671974 rapa rf      (1)   ATGGTGTTGAGGACACAGAGATGGAATCGTCTTACTACTTTGAGATTGGTTCATCTCCGTTCAACTGAGACAGGTACTCT
                            81                                                                            160
KX671969  rapa Rf    (81)   GAGAAATGCTGCTTTCTTCCAAAGCCCATACGACTTCTTCTTCTGCGTACAAGGCTTCTCTGGTCTCACCAGCGATAGAA
KX671968  rapa Rf    (81)   GAGAAATGCTGCTTTCTTCCAAAGCCCATACGACTTCTTCTTCTGCGTACAAGGCTTCTCTGGTCTCACCAGCGATAGAA
KX671967 rapa Rf     (81)   GAGAAATGCTGCTTTCTTCCAAAGCCCATACGACTTCTTCTTCTGCGTACAAGGCTTCTCTGGTCTCACCAGCGATAGAA
EF584011 Rf          (81)   GAGAAATGCTGCTTTCTTCCAAAGCCCATACGAATTCTTCTTCTGCGTACAAGGCTTCTCTGGTCTCACCAGCGATAGAA
KX671970 rapa rf     (81)   GAGAAATGCTGCTTTCTTCCAAAGCCCATACGACTTCTTCTTCTGCGTACAAGGCTTCTCTGGTCTCACCAGCGATAGAA
KX671971  rapa rf    (81)   GAGAAATGCTGCTTTCTTCCAAAGCCCATACGACTTCTTCTTCTGCGTACAAGGCTTCTCTGGTCTCACCAGCGATAGAA
KX671972 rapa rf     (81)   GAGAAATGCTGCTTTCTTCCAAAGCCCATACGACTTCTTCTTCTGCGTACAAGGCTTCTCTGGTCTCACCAGCGATAGAA
KX671973  rapa rf    (81)   GAGAAATGCTGCTTTCTTCCAAAGCCCATACGACTTCTTCTTCTGCGTACAAGGCTTCTCTGGTCTCACCAGCGATAGAA
KX671974 rapa rf     (81)   GAGAAATGCTGCTTTCTTCCAAAGCCCATACGACTTCTTCTTCTGCGTACAAGGCTTCTCTGGTCTCACCAGCGATAGAA
                            161                                                                           240
KX671969  rapa Rf   (161)   AGATGTCTTCTTACAAAGAGAGATTGAGAAGTGGACTCGTCGATATCAAGAAGGATGATGTTGTGGCTCTGTTTCAGTCC
KX671968  rapa Rf   (161)   AGATGTCTTCTTACAAAGAGAGATTGAGAAGTGGACTCGTCGATATCAAGAAGGATGATGTTGTGGCTCTGTTTCAGTCC
KX671967 rapa Rf    (161)   AGATGTCTTCTTACAAAGAGAGATTGAGAAGTGGACTCGTCGATATCAAGAAGGATGATGTTGTGGCTCTGTTTCAGTCC
EF584011 Rf         (161)   AGATGTCTTCTTACAAAGAGAGATTGAGAAGTGGACTCGTCGATATCAAGAAGGATGATGCTGTGGCTCTGTTTCAGTCC
KX671970 rapa rf    (161)   AGATGTCTTCTTACAAAGAGAGATTGAGAAGTGGACTCGTCGATATCAAGAAGGATGATGTTGTGGCTCTGTTTCAGTCC
KX671971  rapa rf   (161)   AGATGTCTTCTTACAAAGAGAGATTGAGAAGTGGTCTCGTCGATATCAAGAAGGATGATGCTGTTGCTCTGTTTCAGTCC
KX671972 rapa rf    (161)   AGATGTCTTCTTACAAAGAGAGATTGAGAAGTGGACTCGTCGATATCAAGAAGGATGATGCTGTGGCTCTGTTTCAGTCC
KX671973  rapa rf   (161)   AGATGTCTTCTTACAAAGAGAGATTGAGAAGTGGTCTCGTCGATATCAAGAAGGATGATGCTGTTGCTCTGTTTCAGTCC
KX671974 rapa rf    (161)   AGATGTCTTCTTACAAAGAGAGATTGAGAAGTGGACTCGTCGATATCAAGAAGGATGATGTTGTGGCTCTGTTTCAGTCC
                            241                                                                           320
KX671969  rapa Rf   (241)   ATGCTTCGGTCTCGTCCTCTTCCTACGGTCATTGATTTCAACAGATTGTTTGGTTTACTTGCCAGAACTAAACAGTACGA
KX671968  rapa Rf   (241)   ATGCTTCGGTCTCGTCCTCTTCCTACGGTCATTGATTTCAACAGATTGTTTGGTTTACTTGCCAGAACTAAACAGTACGA
KX671967 rapa Rf    (241)   ATGCTTCGGTCTCGTCCTCTTCCTACGGTCATTGATTTCAACAGATTGTTTGGTTTACTTGCCAGAACTAAACAGTACGA
EF584011 Rf         (241)   ATGCTTCGGTCTCGTCCTCTTCCTACGGTCATTGATTTCAACAGATTGTTTGGTTTACTTGCCAGAACTAAACAGTACGA
KX671970 rapa rf    (241)   ATGCTTCGGTCTCGTCCTCTTCCTACGGTCATTGATTTCAACAGATTGTTTGGTTTACTTGCCAGAACTAAACAGTACGA
KX671971  rapa rf   (241)   ATGCTTCGGTCTCGTCCTCTTCCTACGGTCATTGATTTCAACAGATTGTTTGGTTTACTTGCCAGAACTAAACAGTACGA
KX671972 rapa rf    (241)   ATGCTTCGGTCTCGTCCTCTTCCTACGGTCATTGATTTCAACAGATTGTTTGGTTTACTTGCCAGAACTAAACAGTACGA
KX671973  rapa rf   (241)   ATGCTTCGGTCTCGTCCTCTTCCTACGGTCATTGATTTCAACAGATTGTTTGGTTTACTTGCCAGAACTAAACAGTACGA
KX671974 rapa rf    (241)   ATGCTTCGGTCTCGTCCTCTTCCTACGGTCATTGATTTCAACAGATTGTTTGGTTTACTTGCCAGAACTAAACAGTACGA
                            321                                                                           400
KX671969  rapa Rf   (321)   TCTCGTGTTAGCTCTCTGCAAGCAAATGGAACTGAAAGGGATTGCGTATGACCTCTACACTCTCAACATTATGATCAATT
KX671968  rapa Rf   (321)   TCTCGTGTTAGCTCTCTGCAAGCAAATGGAACTGAAAGGGATTGCGTATGACCTCTACACTCTCAACATTATGATCAATT
KX671967 rapa Rf    (321)   TCTCGTGTTAGCTCTCTGCAAGCAAATGGAACTGAAAGGGATTGCGTATGACCTCTACACTCTCAACATTATGATCAATT
EF584011 Rf         (321)   TCTCGTGTTAGCTCTCTGCAAGCAAATGGAACTGAAAGGGATTGCGTATGACCTCTACACTCTCAACATTATGATCAATT
KX671970 rapa rf    (321)   TCTCGTGTTAGCTCTCTGCAAGCAAATGGAACTGAAAGGGATTGCGTATGACCTCTACACTCTCAACATTATGATCAATT
KX671971  rapa rf   (321)   TCTCGTGTTAGCTCTCTGCAAGCAAATGGAACTGAAAGGGATTGCGTATGACCTCTACACTCTCAACATTATGATCAATT
KX671972 rapa rf    (321)   TCTCGTGTTAGCTCTCTGCAAGCAAATGGAACTGAAAGGGATTGCGTATGACCTCTACACTCTCAACATTATGATCAATT
KX671973  rapa rf   (321)   TCTCGTGTTAGCTCTCTGCAAGCAAATGGAACTGAAAGGGATTGCGTATGACCTCTACACTCTCAACATTATGATCAATT
KX671974 rapa rf    (321)   TCTCGTGTTAGCTCTCTGCAAGCAAATGGAACTGAAAGGGATTGCGTATGACCTCTACACTCTCAACATTATGATCAATT
                            401                                                                           480
KX671969  rapa Rf   (401)   GCTTCTGCAGGCGTCGGAAACTCGGTTTTGCTTTTTCTGCTATGGGGAAGATCTTCAAACTTGGGTATGAGCCTAACACA
KX671968  rapa Rf   (401)   GCTTCTGCAGGCGTCGGAAACTCGGTTTTGCTTTTTCTGCTATGGGGAAGATCTTCAAACTTGGGTATGAGCCTAACACA
KX671967 rapa Rf    (401)   GCTTCTGCAGGCGTCGGAAACTCGGTTTTGCTTTTTCCGCTATGGGGGAGATCTTCAAACTTGGGTATGAGCCTAACACA
EF584011 Rf         (401)   GCTTCTGCAGGCGTCGGAAACTCGGTTTTGCTTTTTCTGCTATGGGGAAGATCTTCAAACTTGGGTATGAGCCTAACACA
KX671970 rapa rf    (401)   GCTTCTGCAGGCGTCGGAAACTCGGTTTTGCTTTTTCCGCTATGGGGGAGATCTTCAAACTTGGGTATGAGCCTAACACA
KX671971  rapa rf   (401)   GCTTCTGCAGGCGTCGGAAACTCGGTTTTGCTTTTTCCGCTATGGGGGAGATCTTCAAACTTGGGTATGAGCCTAACACA
KX671972 rapa rf    (401)   GCTTCTGCAGGCGTCGGAAACTCGGTTTTGCTTTTTCTGCTATGGGGAAGATCTTCAAACTTGGGTATGAGCCTAACACA
KX671973  rapa rf   (401)   GCTTCTGCAGGCGTCGGAAACTCGGTTTTGCTTTTTCCGCTATGGGGGAGATCTTCAAACTTGGGTATGAGCCTAACACA
KX671974 rapa rf    (401)   GCTTCTGCAGGCGTCGGAAACTCGGTTTTGCTTTTTCCGCTATGGGGGAGATCTTCAAACTTGGGTATGAGCCTAACACA
                            481                                                                           560
KX671969  rapa Rf   (481)   GTCACATTTAACACCCTCCTCAATGGCTTATGTCTCGAGGGCAGAGTCTTTGAAGCTGTGGAGTTAGTTGATTGTATGGT
KX671968  rapa Rf   (481)   GTCACATTTAACACCCTCCTCAATGGCTTATGTCTCGAGGGCAGAGTCTTTGAAGCTGTGGAGTTAGTTGATTGTATGGT
KX671967 rapa Rf    (481)   GTCACATTTAACACCCTCCTCAATGGCTTATGTCTCGAGGGCAGAGTCTTTGAAGCTGTGGAGTTAGTTGATTGTATGGT
EF584011 Rf         (481)   GTCACATTTAACACCCTCCTCAATGGCTTATGTCTCGAGGGCAGAGTCTTTGAAGCTGTGGAGTTAGTTGATTGTATGGT
KX671970 rapa rf    (481)   GTCACATTTAACACCCTCCTCAATGGCTTATGTCTCGAGGGCAGAGTCTTTGAAGCTGTGGAGTTAGTTGATTGTATGGT
KX671971  rapa rf   (481)   GTCACATTTAACACCCTCCTCAATGGCTTATGTCTCGAGGGCAGAGTCTTTGAAGCTGTGGAGTTAGTTGATTGTATGGT
KX671972 rapa rf    (481)   GTCACATTTAACACCCTCCTCAATGGCTTATGTCTCGAGGGCAGAGTCTTTGAAGCCGTGGAGTTAGTTGATTGTATGGT
KX671973  rapa rf   (481)   GTCACATTTAACACCCTCCTCAATGGCTTATGTCTCGAGGGCAGAGTCTTTGAAGCTGTGGAGTTAGTTGATTGTATGGT
```

```
KX671974 rapa rf   (481) GTCACATTTAACACCCTCCTCAATGGCTTATGTCTCGAGGGCAGAGTCTTTGAAGCTGTGGAGTTAGTTGATTGTATGGT
                         561                                                                          640
KX671969  rapa Rf  (561) CCTAAGCCAACATGTACCAGATCTCATCACCCTCAACACTATTGTCAATGGGCTTTGTCTCAAAGATAGAGTGTCTGAAG
KX671968  rapa Rf  (561) CCTAAGCCAACATGTACCAGATCTCATCACCCTCAACACTATTGTCAATGGGCTTTGTCTCAAAGATAGAGTGTCTGAAG
KX671967 rapa Rf   (561) CCTAAGCCAACATGTACCAGATCTCATCACCCTCAACACTCTTGTCAATGGGCTTTGTCTCAAAGATAGAGTGTCTGAAG
     EF584011 Rf   (561) CCTAAGCCAACATGTACCAGATCTCATCACCCTCAACACTATTGTCAATGGGCTTTGTCTCAAAGATAGAGTGTCTGAAG
KX671970 rapa rf   (561) CCTAAGCCAACATGTACCAGATCTCATCACCCTCAACACTCTTGTCAATGGGCTTTGTCTCAAAGATAGAGTGTCTGAAG
KX671971  rapa rf  (561) CCTAAGCCAACATGTACCAGATCTCATCACCCTCAACACTCTTGTCAATGGGCTTTGTCTCAAAGATAGAGTGTCTGAAG
KX671972 rapa rf   (561) CCTAAGCCAACATGTACCAGATCTCATCACCCTCAACACTATTGTCAATGGGCTTTGTCTCAAAGATAGAGTGTCTGAAG
KX671973  rapa rf  (561) CCTAAGCCAACATGTACCAGATCTTATCACCCTCAACACTATTGTCAATGGGCTTTGTCTCAAAGATAGAGTGTCTGAAG
KX671974 rapa rf   (561) CCTAAGCCAACATGTACCAGATCTCATCACCCTCAACACTCTTGTCAATGGGCTTTGTCTCAAAGATAGAGTGTCTGAAG
                         641                                                                          720
KX671969  rapa Rf  (641) CAGTGGATTTAATAGCTCGAATGATGGCTAATGGATGTCAACCCAATCAGTTTACCTATGGTCCAATCTTGAACAGAATG
KX671968  rapa Rf  (641) CAGTGGATTTAATAGCTCGAATGATGGCTAATGGATGTCAACCCAATCAGTTTACCTATGGTCCAATCTTGAACAGAATG
KX671967 rapa Rf   (641) CAGTGGATTTAATAGCTCGAATGATGGCTAATGGATGTCAACCCAATCAGTTTACCTATGGTCCAATCTTGAACAGAATG
     EF584011 Rf   (641) CAGTGGATTTAATAGCTCGAATGATGGCTAATGGATGTCAACCCAATCAGTTTACCTATGGTCCAATCTTGAACAGAATG
KX671970 rapa rf   (641) CAGTGGATTTAATAGCTCGAATGATGGCTAATGGATGTCAACCCAATCAGTTTACCTATGGTCCAATCTTGAACAGAATG
KX671971  rapa rf  (641) CAGTGGATTTAATAGCTCGAATGATGGCTAATGGATGTCAACCCAATCAGTTTACCTATGGTCCAATCTTGAACAGAATG
KX671972 rapa rf   (641) CAGTGGATTTAATAGCTCGAATGATGGATAAAGGATGTCAAGCCGATCAGTTTACCTATGGTCCAATCTTGAACAGAATG
KX671973  rapa rf  (641) CAGTGGATTTAATAGCTCGAATGATGGATAAAGGATGTCAAGCCGATCAGTTTACCTATGGTCCAATCTTGAACAGAATG
KX671974 rapa rf   (641) CAGTGGATTTAATAGCTCGAATGATGGCTAATGGATGTCAACCCAATCAGTTTACCTATGGTCCAATCTTGAACAGAATG
                         721                                                                          800
KX671969  rapa Rf  (721) TGTAAGTCTGGGAACACTGCCTCGGCCTTGGATCTGCTCAGGAAGATGGAACATAGAAAGATCAAGCCACACGTAGTCAC
KX671968  rapa Rf  (721) TGTAAGTCTGGGAACACTGCCTCGGCCTTGGATCTGCTCAGGAAGATGGAACATAGAAAGATCAAGCCACACGTAGTCAC
KX671967 rapa Rf   (721) TGTAAGTCTGGGAACACTGCCTCGGCCTTGGATCTGCTCAGGAAGATGGAACATAGAAAGATCAAGCCACACGTAGTCAC
     EF584011 Rf   (721) TGTAAGTCTGGGAACACTGCCTCGGCCTTGGATCTGCTCAGGAAGATGGAACATAGAAAGATCAAGCCACACGTAGTCAC
KX671970 rapa rf   (721) TGTAAGTCTGGGAACACTGCCTCGGCCTTGGATCTGCTCAGGAAGATGGAACATAGAAAGATCAAGCCACACGTAGTCAC
KX671971  rapa rf  (721) TGTAAGTCTGGGAACACTGCCTCGGCCTTGGATCTGCTCAGGAAGATGGAACATAGAAAGATCAAGCCACACGTAGTCAC
KX671972 rapa rf   (721) TGTAAGTCTGGGAACACTGCCTCGGCCTTGGATCTGCTCAGGAAGATGGAACATAGAAAGATCAAGCCACACGTAGTCAC
KX671973  rapa rf  (721) TGTAAGTCTGGGAACACTGCCTCGGCCTTGGATCTGCTCAGGAAGATGGAACATAGAAAGATCAAGCCACACGTAGTCAC
KX671974 rapa rf   (721) TGTAAGTCTGGGAACACTGCCTCGGCCTTGGATCTGCTCAGGAAGATGGAACATAGAAAGATCAAGCCACACGTAGTCAC
                         801                                                                          880
KX671969  rapa Rf  (801) ATACACTATCATCATTGACAATCTTTGCAAAGATGGGAGACTCGACGATGCACTCAGCTTTTTCAGTGAAATGGAAACCA
KX671968  rapa Rf  (801) ATACACTATCATCATTGACAATCTTTGCAAAGATGGGAGACTCGACGATGCACTCAGCTTTTTCAGTGAAATGGAAACCA
KX671967 rapa Rf   (801) ATACACTATCATCATTGACAATCTTTGCAAAGATGGGAGACTCGACGATGCACTCAGCTTTTTCAGTGAAATGGAAACCA
     EF584011 Rf   (801) ATACACTATCATCATTGACAATCTTTGCAAAGATGGGAGACTCGACGATGCACTCAGCTTTTTCAGTGAAATGGAAACCA
KX671970 rapa rf   (801) ATACACTATCATCATTGACAATCTTTGCAAAGATGGGAGACTCGACGATGCACTCAGCTTTTTCAGTGAAATGGAAACCA
KX671971  rapa rf  (801) ATACACTATCATCATTGACAATCTTTGCAAAGATGGGAGACTCGACGATGCACTCAGCTTTTTCAGTGAAATGGAAACCA
KX671972 rapa rf   (801) ATACAATATCATCATTGACAATCTTTGCAAAGATGGGAGACTCGACGATGCACTCAGCTTTTTCAGTGAAATGGAAACCA
KX671973  rapa rf  (801) ATACAATATCATCATTGACAATCTTTGCAAAGATGGGAGACTCGACGATGCACTCAGCTTTTTCAGTGAAATGGAAACCA
KX671974 rapa rf   (801) ATACAATATCATCATTGACAATCTTTGCAAAGATGGGAGACTCGACGATGCACTCAGCTTTTTCAGTGAAATGGAAACCA
                         881                                                                          960
KX671969  rapa Rf  (881) AAGGGATCAAAGCAAATGTCTTTACCTACAACTCTCTCATAGGAAGCTTCTGTAGTTTTGGCAGATGGGATGATGGTGCA
KX671968  rapa Rf  (881) AAGGGATCAAAGCAAATGTCTTTACCTACAACTCTCTCATAGGAAGCTTCTGTAGTTTTGGCAGATGGGATGATGGTGCA
KX671967 rapa Rf   (881) AAGGGATCAAAGCAAATGTCTTTACCTACAACTCTCTCATAGGAAGCTTCTGTAGTTTTGGCAGATGGGATGATGGTGCA
     EF584011 Rf   (881) AAGGGATCAAAGCAAATGTCTTTACCTACAACTCTCTCATAGGAAGCTTCTGTAGTTTTGGCAGATGGGATGATGGTGCA
KX671970 rapa rf   (881) AAGGGATCAAAGCAAATGTCTTTACCTACAACTCTCTCATAGGAAGCTTCTGTAGTTTTGGCAGATGGGATGATGGTGCA
KX671971  rapa rf  (881) AAGGGATCAAAGCAAATGTCTTTACCTACAACTCTCTCATAGGAAGCTTCTGTAGTTTTGGCAGATGGGATGATGGTGCA
KX671972 rapa rf   (881) AAGGGATCAAAGCAGATGTCATTACCTACAACTCTCTCATAGGAAGCTTCTGTAGTTTTGGCAGATGGGATGATGGTGCA
KX671973  rapa rf  (881) AAGGGATCAAAGCAGATGTCATTACCTACAACTCTCTCATAGGAAGCTTCTGTAGTTTTGGCAGATGGGATGATGGTGCA
KX671974 rapa rf   (881) AAGGGATCAAAGCAGATGTCATTACCTACAACTCTCTCATAGGAAGCTTCTGTAGTTTTGGCAGATGGGATGATGGTGCA
                         961                                                                         1040
KX671969  rapa Rf  (961) CAGTTGCTGAGGGATATGATTACAAGGAAAATCACCCCCAACGTTGTCACTTTCAGTGCTTTGATTGATAGTCTTGTTAA
KX671968  rapa Rf  (961) CAGTTGCTGAGGGATATGATTACAAGGAAAATCACCCCCAACGTTGTCACTTTCAGTGCTTTGATTGATAGTCTTGTTAA
KX671967 rapa Rf   (961) CAGTTGCTGAGGGATATGATTACAAGGAAAATCACCCCCAACGTTGTCACTTTCAGTGCTTTGATTGATAGTCTTGTTAA
     EF584011 Rf   (961) CAGTTGCTGAGGGATATGATTACAAGGAAAATCACCCCCAACGTTGTCACTTTCAGTGCTTTGATTGATAGTCTTGTTAA
KX671970 rapa rf   (961) CAGTTGCTGAGGGATATGATTACAAGGAAAATCACCCCCAACGTTGTCACTTTCAGTGCTTTGATTGATAGTCTTGTTAA
KX671971  rapa rf  (961) CAGTTGCTGAGGGATATGATTACAAGGAAAATCACCCCCAACGTTGTCACTTTCAGTGCTTTGATTGATAGTCTTGTTAA
KX671972 rapa rf   (961) CAGTTGCTGAGGGATATGATTACAAGGAAAATCACCCCCAACGTTGTCACTTTCAGTGCTTTGATTGATAGTCTTGTTAA
KX671973  rapa rf  (961) CAGTTGCTGAGGGATATGATTACAAGGAAAATCACCCCCAACGTTGTCACTTTCAGTGCTTTGATTGATAGTCTTGTTAA
KX671974 rapa rf   (961) CAGTTGCTGAGGGATATGATTACAAGGAAAATCACCCCCAACGTTGTCACTTTCAGTGCTTTGATTGATAGTCTTGTTAA
                         1041                                                                        1120
KX671969  rapa Rf (1041) AGAGGGAAAGCTTACTGAGGCTAAAGACTTGTACAATGAGATGATCACAAGAGGCATAGAGCCTAACACCATTACATATA
KX671968  rapa Rf (1041) AGAGGGAAAGCTTACTGAGGCTAAAGACTTGTACAATGAGATGATCACAAGAGGCATAGAGCCTAACACCATTACATATA
KX671967 rapa Rf  (1041) AGAGGGAAAGCTTACTGAGGCTAAAGACTTGTACAATGAGATGATCACAAGAGGCATAGAGCCTAACACCATTACATATA
     EF584011 Rf  (1041) AGAGGGAAAGCTTACTGAGGCTAAAGACTTGTACAATGAGATGATCACAAGAGGCATAGAGCCTAACACCATTACATATA
KX671970 rapa rf  (1041) AGAGGGAAAGCTTACTGAGGCTAAAGACTTGTACAATGAGATGATCACAAGAGGCATAGAGCCTAACACCATTACATATA
KX671971  rapa rf (1041) AGAGGGAAAGCTTACTGAGGCTAAAGACTTGTACAATGAGATGATCACAAGAGGCATAGAGCCTAACACCATTACATATA
KX671972 rapa rf  (1041) AGAGGGAAAGCTTACTGAGGCTAAAGACTTGTACAATGAGATGATCACAAGAGGCATAGATCCTAATACCATTACATATA
```

```
KX671973  rapa rf  (1041) AGAGGGAAAGCTTACTGAGGCTAAAGACTTGTACAATGAGATGATCACAAGAGGCATAGATCCTAATACCATTACATATA
 KX671974 rapa rf  (1041) AGAGGGAAAGCTTACTGAGGCTAAAGACTTGTACAATGAGATGATCACAAGAGGCATAGATCCTAATACCATTACATATA
                          1121                                                                        1200
KX671969  rapa Rf  (1121) ATTCTTTGATATATGGGCTGTGCAACGACAAGCGCTTAGATGAAGCCAACCAGATGATGGACCTGATGGTTAGCAAGGGA
KX671968  rapa Rf  (1121) ATTCTTTGATATATGGGCTGTGCAACGACAAGCGCTTAGATGAAGCCAACCAGATGATGGACCTGATGGTTAGCAAGGGA
 KX671967 rapa Rf  (1121) ATTCTTTGATATATGGGCTGTGCAACGACAAGCGCTTAGATGAAGCCAACCAGATGATGGACCTGATGGTTAGCAAGGGA
      EF584011 Rf  (1121) ATTCTTTGATATATGGGCTGTGCAACGACAAGCGCTTAGATGAAGCCAACCAGATGATGGACCTGATGGTTAGCAAGGGA
 KX671970 rapa rf  (1121) ATTCTTTGATATATGGGCTGTGCAACGACAAGCGCTTAGATGAAGCCAACCAGATGATGGACCTGATGGTTAGCAAGGGA
KX671971  rapa rf  (1121) ATTCTTTGATATATGGGCTGTGCAACGACAAGCGCTTAGATGAAGCCAACCAGATGATGGACCTGATGGTTAGCAAGGGA
 KX671972 rapa rf  (1121) GTACTTTGATATATGGGTTGTGCATGGAGAACCGCTTAGATGAAGCCAACCAGATGATGGACCTCATGGTTAGCAAGGGA
KX671973  rapa rf  (1121) GTACTTTGATATATGGGTTGTGCATGGAGAACCGCTTAGATGAAGCCAACCAGATGATGGACCTCATGGTTAGCAAGGGA
 KX671974 rapa rf  (1121) GTACTTTGATATATGGGTTGTGCATGGAGAACCGCTTAGATGAAGCCAACCAGATGATGGACCTCATGGTTAGCAAGGGA

                          1201                                                                        1280
KX671969  rapa Rf  (1201) TGCGATCCTGATATTTGGACGTATAATATCCTTATAAACGGATTTTGTAAGGCTAAACAGGTTGATGATGGTATGAGACT
KX671968  rapa Rf  (1201) TGCGATCCTGATATTTGGACGTATAATATCCTTATAAACGGATTTTGTAAGGCTAAACAGGTTGATGATGGTATGAGACT
 KX671967 rapa Rf  (1201) TGCGATCCTGATATTTGGACGTATAATATCCTTATAAACGGATTTTGTAAGGCTAAACAGGTTGATGATGGTATGAGACT
      EF584011 Rf  (1201) TGCGATCCTGATATTTGGACGTATAATATCCTTATAAACGGATTTTGTAAGGCTAAACAGGTTGATGATGGTATGAGACT
 KX671970 rapa rf  (1201) TGCGATCCTGATATCGTGACTTTTAATGTCCTTATAAACGGATTTTGTAAGGCTAAACAGGTTGATGATGGTATGAGACT
KX671971  rapa rf  (1201) TGCGATCCTGATATCGTGACTTTTAATGTCCTTATAAACGGATTTTGTAAGGCTAAACAGGTTGATGATGGTATGAGACT
 KX671972 rapa rf  (1201) TGCGATCCTGATATCGTGACGTTTAATGTCCTTATAAACGGATTTTGTAAGGCTAAACAGGTTGATGTTGGTATGAGACT
KX671973  rapa rf  (1201) TGCGATCCTGATATCGTGACGTTTAATGTCCTTATAAACGGATTTTGTAAGGCTAAACAGGTTGATGTTGGTATGAGACT
 KX671974 rapa rf  (1201) TGCGATCCTGATATCGTGACGTTTAATGTCCTTATAAACGGATTTTGTAAGGCTAAACAGGTTGATGTTGGTATGAGACT
                          1281                                                                        1360
KX671969  rapa Rf  (1281) ATTCCGAAAGATGTCTCTGAGAGGAATGATTGCAGATACAGTGACTTATAGCACTCTCATCCAAGGGTTTTGTCAATCAA
KX671968  rapa Rf  (1281) ATTCCGAAAGATGTCTCTGAGAGGAATGATTGCAGATACAGTGACTTATAGCACTCTCATCCAAGGGTTTTGTCAATCAA
 KX671967 rapa Rf  (1281) ATTCCGAAAGATGTCTCTGAGAGGAATGATTGCAGATACAGTGACTTATAGCACTCTCATCCAAGGGTTTTGTCAATCAA
      EF584011 Rf  (1281) ATTCCGAAAGATGTCTCTGAGAGGAATGATTGCAGATACAGTGACTTATAGCACTCTCATCCAAGGGTTTTGTCAATCAA
 KX671970 rapa rf  (1281) ATTCCGAAAGATGTCTCTGAGAGGAATGATTGCAGATACAGTGACTTATAGCACTCTCATCCAAGGGTTTTGTCAATCAA
KX671971  rapa rf  (1281) ATTCCGAAAGATGTCTCTGAGAGGAATGATTGCAGATACAGTGACTTATAGCACTCTCATCCAAGGGTTTTGTCAATCAA
 KX671972 rapa rf  (1281) ATTCCGAAAGATGTCTCTGAGAGGAGTGATTGCAGATACAGTGACTTATAGCACTCTCATCCAAGGGTTTTGTCAATCAA
KX671973  rapa rf  (1281) ATTCCGAAAGATGTCTCTGAGAGGAGTGATTGCAGATACAGTGACTTATAGCACTCTCATCCAAGGGTTTTGTCAATCAA
 KX671974 rapa rf  (1281) ATTCCGAAAGATGTCTCTGAGAGGAGTGATTGCAGATACAGTGACTTATAGCACTCTCATCCAAGGGTTTTGTCAATCAA
                          1361                                                                        1440
KX671969  rapa Rf  (1361) GAAAACTTATTGTCGCCAAAAAAGTCTTCCAAGAGATGGTCTCTCAAGGTGTTCATCCTGGTATTATGACTTATGCTATT
KX671968  rapa Rf  (1361) GAAAACTTATTGTCGCCAAAAAAGTCTTCCAAGAGATGGTCTCTCAAGGTGTTCATCCTGGTATTATGACTTATGCTATT
 KX671967 rapa Rf  (1361) GAAAACTTATTGTCGCCAAAAAAGTCTTCCAAGAGATGGTCTCTCAAGGTGTTCATCCTGGTATTATGACTTATGCTATT
      EF584011 Rf  (1361) GAAAACTTATTGTCGCCAAAAAAGTCTTCCAAGAGATGGTCTCTCAAGGTGTTCATCCTGGTATTATGACTTATGCTATT
 KX671970 rapa rf  (1361) GAAAACTTATTGTCGCCAAAAAAGTCTTCCAAGAGATGGTCTCTCAAGGTGTTCATCCTGGTATTATGACTTATGCTATT
KX671971  rapa rf  (1361) GAAAACTTATTGTCGCCAAAAAAGTCTTCCAAGAGATGGTCTCTCAAGGTGTTCATCCTGGTATTATGACTTATGCTATT
 KX671972 rapa rf  (1361) GAGAACTTATTGTCGCCAAAGAAGTCTTCCAAGAGATGGTCTCTCAAGGTGTTCATCCTGGTATTATGACTTATGGTATT
KX671973  rapa rf  (1361) GAGAACTTATTGTCGCCAAAGAAGTCTTCCAAGAGATGGTCTCTCAAGGTGTTCATCCTGGTATTATGACTTATGGTATT
 KX671974 rapa rf  (1361) GAGAACTTATTGTCGCCAAAGAAGTCTTCCAAGAGATGGTCTCTCAAGGTGTTCATCCTGGTATTATGACTTATGGTATT
                          1441                                                                        1520
KX671969  rapa Rf  (1441) TTGCTGGATGGGTTGTGTGACAATGGCGAACTAGAAGAGGCTTTGGGAATACTTGATCAAATGCACAAGTGTAAGATGGA
KX671968  rapa Rf  (1441) TTGCTGGATGGGTTGTGTGACAATGGCGAACTAGAAGAGGCTTTGGGAATACTTGATCAAATGCACAAGTGTAAGATGGA
 KX671967 rapa Rf  (1441) TTGCTGGATGGGTTGTGTGACAATGGCGAACTAGAAGAGGCTTTGGGAATACTTGATCAAATGCACAAGTGTAAGATGGA
      EF584011 Rf  (1441) TTGCTGGATGGGTTGTGTGACAATGGCGAACTAGAAGAGGCTTTGGGAATACTTGATCAAATGCACAAGTGTAAGATGGA
 KX671970 rapa rf  (1441) TTGCTGGATGGGTTGTGTGACAATGGCGAACTAGAAGAGGCTTTGGGAATACTTGATCAAATGCACAAGTGTAAGATGGA
KX671971  rapa rf  (1441) TTGCTGGATGGGTTGTGTGACAATGGCGAACTAGAAGAGGCTTTGGGAATACTTGATCAAATGCACAAGTGTAAGATGGA
 KX671972 rapa rf  (1441) TTGCTGGATGGGTTGTGTGACAATGGCGAACTAGAAGAGGCTTTGGGAATACTTGATCAAATGCACAAGTGTAAGATGGA
KX671973  rapa rf  (1441) TTGCTGGATGGGTTGTGTGACAATGGCGAACTAGAAGAGGCTTTGGGAATACTTGATCAAATGCACAAGTGTAAGATGGA
 KX671974 rapa rf  (1441) TTGCTGGATGGGTTGTGTGACAATGGCGAACTAGAAGAGGCTTTGGGAATACTTGATCAAATGCACAAGTGTAAGATGGA
                          1521                                                                        1600
KX671969  rapa Rf  (1521) ACTTGATATTGGTATATATAAATATCATCATTCACGGGATGTGCAATGCAAATAAGGTCGATGATGCTTGGAGTTTGTTCT
KX671968  rapa Rf  (1521) ACTTGATATTGGTATATATAAATATCATCATTCACGGGATGTGCAATGCAAATAAGGTCGATGATGCTTGGAGTTTGTTCT
 KX671967 rapa Rf  (1521) ACTTGATATTGGTATATATAAATATCATCATTCACGGGATGTGCAATGCAAATAAGGTCGATGATGCTTGGAGTTTGTTCT
      EF584011 Rf  (1521) ACTTGATATTGGTATATATAAATATCATCATTCACGGGATGTGCAATGCAAATAAGGTCGATGATGCTTGGAGTTTGTTCT
 KX671970 rapa rf  (1521) ACTTGATATTGGTATATATAAATATCATCATTCACGGGATGTGCAATGCAAATAAGGTCGATGATGCTTGGAGTTTGTTCT
KX671971  rapa rf  (1521) ACTTGATATTGGTATATATAAATATCATCATTCACGGGATGTGCAATGCAAATAAGGTCGATGATGCTTGGAGTTTGTTCT
 KX671972 rapa rf  (1521) ACTTGATATTGGTATATATAGTATCATCATTCATGGGTTGTGCAATGCAAGTAAGATCGATGATGCTTGGGATCTATTCT
KX671973  rapa rf  (1521) ACTTGATATTGGTATATATAGTATCATCATTCACGGGTTGTGCAATGCAAGTAAGATCGATGATGCTTGGGATCTATTCT
 KX671974 rapa rf  (1521) ACTTGATATTGGTATATATAGTATCATCATTCACGGGTTGTGCAATGCAAGTAAGATCGATGATGCTTGGGATCTATTCT
                          1601                                                                        1680
KX671969  rapa Rf  (1601) GTAGCCTCTCTCAAAGGAGTGAAGCGTGATATTCAGTCATACAACATAATGTTGTCAGGATTATGTAAGAGGAGCTCA
KX671968  rapa Rf  (1601) GTAGCCTCTCTCAAAGGAGTGAAGCGTGATATTCAGTCATACAACATAATGTTGTCAGGATTATGTAAGAGGAGCTCA
 KX671967 rapa Rf  (1601) GTAGCCTCTCTCAAAGGAGTGAAGCGTGATATTCAGTCATACAACATAATGTTGTCAGGATTATGTAAGGGGAGCTCA
      EF584011 Rf  (1601) GTAGCCTACCTTCGAAAGGAGTGAAGCGTGATATTCAGTCATACAACATAATGTTGTCAGGATTATGTAAGAGGAGCTCA
 KX671970 rapa rf  (1601) GTAGCCTACCTTCGAAAGGAGTGAAGCGTGATATTCAGTCATACAACATAATGTTGTCAGGATTATGTAAGAGGAGCTCA
```

```
KX671971   rapa rf   (1601) GTAGCCTACCTTCGAAAGGAGTGAAGCGTGATATTCAGTCATACAACATAATGTTGTCAGGATTATGTAAGAGGAGCTCA
 KX671972 rapa rf   (1601) GTAGCCTCTCTCTCAAAGGAGTGAAGCGTGATATTCAGTCATACAACATAATGTTGTCAGGATTATGTAAAAGGAGCTCA
KX671973   rapa rf   (1601) GTAGCCTCTCTCTCAAAGGAGTGAAGCGTGATATTCAGTCATACAACATAATGTTGTCAGGATTATGTAAAAGGAGCTCA
 KX671974 rapa rf   (1601) GTAGCCTCTCTCTCAAAGGAGTGAAGCGTGATATTCAGTCATACAACATAATGTTGTCAGGATTATGTAAAAGGAGCTCA
                            1681                                                                        1760
KX671969   rapa Rf   (1681) TTGTCTGAAGCGGATGCATTGTTTAGAAAAATGAAGGAAGATGGGTATGAGCCAGATGGTTGTACGTACAATACACTTAT
KX671968   rapa Rf   (1681) TTGTCTGAAGCGGATGCATTGTTTAGAAAAATGAAGGAAGATGGGTATGAGCCAGATGGTTGTACGTACAATACACTTAT
 KX671967 rapa Rf   (1681) TTGTCTGAAGCGGATGCATTGTTTAGAAAAATGAAGGAAGATGGGTATGAGCCAGATGGTTGTACGTACAATACACTTAT
      EF584011 Rf   (1681) TTGTCTGAAGCGGATGCATTGTTTAGAAAAATGAAGGAAGATGGGTATGAGCCAGATGGTTGTACGTACAATACACTTAT
 KX671970 rapa rf   (1681) TTGTCTGAAGCGGATGCATTGTTTAGAAAAATGAAGGAAGATGGGTATGAGCCAGATGGTTGTACGTACAATACACTTAT
KX671971   rapa rf   (1681) TTGTCTGAAGCGGATGCATTGTTTAGAAAAATGAAGGAAGATGGGTATGAGCCAGATGGTTGTACGTACAATACACTTAT
 KX671972 rapa rf   (1681) TTGTCTGAAGCGGATGCATTGTTTAGAAAAATGAAGGAAGATGGGTATGAGCCAGATGGTTGTACGTACAATACACTTAT
KX671973   rapa rf   (1681) TTGTCTGAAGCGGATGCATTGTTTAGAAAAATGAAGGAAGATGGGTATGAGCCAGATGGTTGTACGTACAATACACTTAT
 KX671974 rapa rf   (1681) TTGTCTGAAGCGGATGCATTGTTTAGAAAAATGAAGGAAGATGGGTATGAGCCAGATGGTTGTACGTACAATACACTTAT
                            1761                                                                        1840
KX671969   rapa Rf   (1761) CAGAGCACATCTTCGAGGTAATGACATAACAACTTCAGTTCAACTCATTGAAGAAATGAAGAGGTGTGGGTTCTCTTCAG
KX671968   rapa Rf   (1761) CAGAGCACATCTTCGAGGTAATGACATAACAACTTCAGTTCAACTCATTGAAGAAATGAAGAGGTGTGGGTTCTCTTCAG
 KX671967 rapa Rf   (1761) CAGAGCACATCTTCGAGGTAATGACATAACAACTTCAGTTCAACTCATTGAAGAAATGAAGAGGTGTGGGTTCTCTTCAG
      EF584011 Rf   (1761) CAGAGCACATCTTCGAGGTAATGACATAACAACTTCAGTTCAACTCATTGAAGAAATGAAGAGGTGTGGGTTCTCTTCAG
 KX671970 rapa rf   (1761) CAGAGCACATCTTCGAGGTAATGACATAACAACTTCAGTTCAACTCATTGAAGAAATGAAGAGGTGTGGGTTCTCTTCAG
KX671971   rapa rf   (1761) CAGAGCACATCTTCGAGGTAATGACATAACAACTTCAGTTCAACTCATTGAAGAAATGAAGAGGTGTGGGTTCTCTTCAG
 KX671972 rapa rf   (1761) CAGAGCACATCTTCGAGGTAGTGACATAACAACTTCAGTTCAACTCATTGAAGAAATGAAGAGGTGTGGGTTCTCTTCAG
KX671973   rapa rf   (1761) CAGAGCACATCTTCGAGGTAGTGACATAACAACTTCAGTTCAACTCATTGAAGAAATGAAGAGGTGTGGGTTCTCTTCAG
 KX671974 rapa rf   (1761) CAGAGCACATCTTCGAGGTAGTGACATAACAACTTCAGTTCAACTCATTGAAGAAATGAAGAGGTGTGGGTTCTCTTCAG
                            1841                                                                        1920
KX671969   rapa Rf   (1841) ATGCTTCCACCGTAAAGATTGTTATGGATATGTTATCGAGTGGTGAATTGGACAAAAGCTTTCTAAATATGCTTTCTGGT
KX671968   rapa Rf   (1841) ATGCTTCCACCGTAAAGATTGTTATGGATATGTTATCGAGTGGTGAATTGGACAAAAGCTTTCTAAATATGCTTTCTGGT
 KX671967 rapa Rf   (1841) ATGCTTCCACCGTAAAGATTGTTATGGATATGTTATCGAGTGGTGAATTGGACAAAAGCTTTCTAAATATGCTTTCTGGT
      EF584011 Rf   (1841) ATGCTTCCACCGTAAAGATTGTTATGGATATGTTATCGAGTGGTGAATTGGACAAAAGCTTTCTAAATATGCTTTCTGGT
 KX671970 rapa rf   (1841) ATGCTTCCACCGTAAAGATTGTTATGGATATGTTATCGAGTGGTGAATTGGACAAAAGCTTTCTAAATATGCTTTCTGGT
KX671971   rapa rf   (1841) ATGCTTCCACCGTAAAGATTGTTATGGATATGTTATCGAGTGGTGAATTGGACAAAAGCTTTCTAAATATGCTTTCTGGT
 KX671972 rapa rf   (1841) ATGCTTCCACCGTAAAGATTGTTATGGATATGCTATCAAGTGGTGAATTGGACAAAAGCTTTCTAAATATGCTTTCTGGT
KX671973   rapa rf   (1841) ATGCTTCCACCGTAAAGATTGTTATGGATATGCTATCAAGTGGTGAATTGGACAAAAGCTTTCTAAATATGCTTTCTGGT
 KX671974 rapa rf   (1841) ATGCTTCCACCGTAAAGATTGTTATGGATATGCTATCAAGTGGTGAATTGGACAAAAGCTTTCTAAATATGCTTTCTGGT
                            1921                            1953
KX671969   rapa Rf   (1921) CCTTTTGGAGACAAATCATCATCGTTGGATTGA
KX671968   rapa Rf   (1921) CCTTTTGGAGACAAATCATCATCGTTGGATTGA
 KX671967 rapa Rf   (1921) CCTTTTGGAGACAAATCATCATCGTTGGATTGA
      EF584011 Rf   (1921) CCTTTTGGAGACAAATCATCATCGTTGGATTGA
 KX671970 rapa rf   (1921) CCTTTTGGAGACAAATCATCATCGTTGGATTGA
KX671971   rapa rf   (1921) CCTTTTGGAGACAAATCATCATCGTTGGATTGA
 KX671972 rapa rf   (1921) CCTTTTGGAGACAAATCATCATTGTTGGATTGA
KX671973   rapa rf   (1921) CCTTTTGGAGACAAATCATCATTGTTGGATTGA
 KX671974 rapa rf   (1921) CCTTTTGGAGACAAATCATCATTGTTGGATTGA
```

Figure S3. The alignment of *R* and *r* nucleotide acid sequences. 1215-1228 region was the core difference of *R(Rf)* and *r* (*rf*) genes. GenBank accession numbers of restorer (*R*) genes, KX671969, KX671968, KX671967, EF584011; GenBank accession numbers of recessive nuclear sterile (r) genes, KX671970, KX671971, KX671972, KX671973, KX671974.
